# The journey to CAR T cell therapy: the pediatric and young adult experience with relapsed or refractory B-ALL

**DOI:** 10.1038/s41408-018-0164-6

**Published:** 2019-01-22

**Authors:** George Hucks, Susan R. Rheingold

**Affiliations:** 1 Division of Pediatric Hematology/Oncology, University of North Carolina at Chapel Hill, Chapel Hill, NC USA; 2 Division of Oncology, Children’s Hospital of Philadelphia, Philadelphia, PA USA; 3 Perelman School of Medicine, Philadelphia, PA USA

## Abstract

Outcomes of pediatric and young adult patients diagnosed with acute lymphoblastic leukemia (ALL) have improved significantly in the past few decades. Treatment advances have provided 5-year survival rates ranging from 78 to 91% depending on the age at diagnosis. However, approximately 2–3% of patients will present with refractory disease that is unresponsive to chemotherapy, and 10–15% of patients will relapse. Outcomes post-relapse show significantly reduced 5-year survival rates that continue to decrease with each subsequent relapse. Despite our increased understanding of risk factors and disease predictors, treatment strategies for patients with relapsed or refractory (r/r) disease, including variations of chemotherapy and stem cell transplant, remain ineffective for many patients. To improve outcomes of patients with r/r disease, immunotherapies targeting specific B cell antigens are being developed. Tisagenlecleucel is an autologous anti-CD19 chimeric antigen receptor (CAR) T cell therapy recently approved by the US Food and Drug Administration for patients with refractory leukemia or those with second or later relapse. In this treatment strategy, a patient’s own T cells are transduced to express an anti-CD19 CAR that, when reintroduced into the patient, directs specific binding and killing of CD19+ B cells. In a phase 2, single-arm, multicenter, global study, tisagenlecleucel resulted in a remission rate of 81% in pediatric and adolescent patients with r/r B cell ALL. This review article summarizes four typical cases of pediatric and adolescent r/r B-cell ALL, focusing on the patient’s journey from initial diagnosis to treatment with CAR T cell therapy.

## Introduction

Although it can occur at any age, acute lymphoblastic leukemia (ALL) is generally a disease of children and young adults. ALL accounts for 25% of cancers in children <15 years of age and 19% of malignancies in adolescents aged 15–19 years^1,2^. Over the past few decades, 5-year survival rates in children and adolescents up to 19 years of age with ALL have increased substantially—from 31% in 1975 to >90% in the mid-2000s^3–5^. However, approximately 2–3% of patients will present with disease that is refractory to induction chemotherapy^6^, and another 10–15% will experience relapse despite successful initial treatment^5,7,8^. Despite these advances, the prognosis for patients with refractory or relapsed (r/r) ALL has not improved, and recurrent ALL remains the leading cause of cancer-related death in children^8,9^.

Approximately 1 in 5 children and adolescents diagnosed with ALL will have r/r disease and undergo salvage treatment. Risk factors for relapse include high white blood cell (WBC) count at presentation, age <1 or ≥10 years at diagnosis, certain cytogenetic abnormalities, such as Philadelphia chromosome (Ph)-like ALL and t(17;19), Down syndrome, and nonadherence to therapy^1,6^. For children with relapsed disease, second remission rates can vary from approximately 70 to 90%^8,10^, yet 5-year survival rates approximate 30% and are further reduced to 10% after ≥2 relapses^11,12^. Children and young adults with primary refractory disease experience similarly poor outcomes. A meta-analysis of children aged 0–18 years with primary refractory disease estimated 10-year survival to be 32%^6^.

Factors that influence prognosis following relapse include length of first remission and site of recurrence (e.g., bone marrow [BM] or extramedullary). Duration of first remission remains one of the strongest predictors of survival. Early relapse (within 18 months of initial diagnosis) is associated with worse overall survival compared with intermediate (18–36 months) or late (>36 months) relapse^9^. Most relapses occur in the BM, but extramedullary sites, including the central nervous system (CNS) and testes, are involved in 20–25% of patients^9,13,14^. Outcomes of patients with isolated extramedullary disease are slightly more favorable than those of patients with BM relapse. Seventy percent of patients with late relapse isolated to an extramedullary site and 40–50% of patients with early extramedullary relapse respond to treatment^15,16^. Only approximately 50% of patients with late BM relapse and 20–30% of patients with early BM relapse benefit from chemotherapy combination regimens^17^.

For first relapse, multidrug high-dose chemotherapy regimens are the primary treatment strategy^18–20^. Chemotherapy alone, however, is not sufficient to maintain long-term remission in the higher-risk subset of relapsed patients. In these cases, allogeneic hematopoietic stem cell transplant (SCT) is the preferred option for patients who achieve a second complete response (CR) and may improve the prognosis^21,22^. The prognosis for patients who are not eligible for SCT or who relapse following SCT is very poor.

**Fig. 1:**
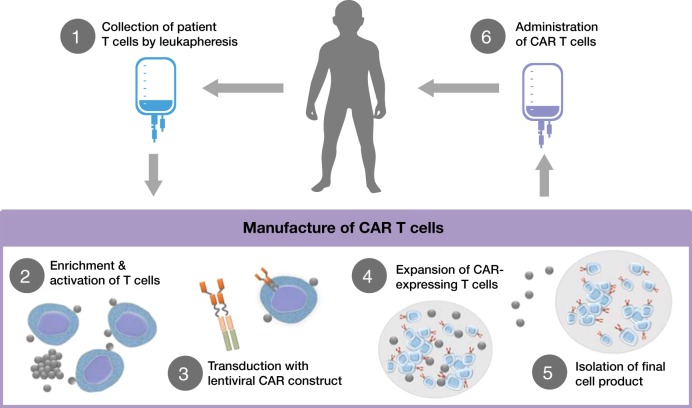
Diagram of CAR T cell treatment process. The treatment process for patients receiving CAR T cell therapy begins with leukapheresis of the patient’s T cells. Once isolated, autologous T cells are sent for manufacturing to produce genetically modified CAR T cells, which are reprogrammed to facilitate targeted killing of CD19+ B cells. The treatment process is completed with intravenous infusion of CAR T cells back to the patient. CAR chimeric antigen receptor

In the past decade, immunotherapies involving endogenous T cells have emerged as a new strategy to treat r/r ALL and avoid chemotherapy resistance. Blinatumomab, a bispecific T cell engager monoclonal antibody that facilitates formation of an immunological synapse between an endogenous T cell receptor and CD19 expressed on B cells, resulted in an overall response rate of 43% in adult patients^23^ and 39% in pediatric patients with r/r ALL^24^. Another approach has been to genetically modify patients’ T cells with a chimeric antigen receptor (CAR) targeting CD19. Briefly, a patient’s T cells are collected via leukapheresis and transduced with a lentiviral construct coding for a CAR. The CAR typically contains a cytoplasmic domain that is active in T cells as well as an extracellular domain that recognizes proteins expressed by B cells. The most studied CAR targets the B cell protein CD19. Once the patient’s T cells express the CAR construct, they are returned to the patient. Following reinfusion, the CAR directs the patient’s T cells to bind to and destroy B cells expressing CD19 (Fig. 1). The CAR T cell therapy tisagenlecleucel (formerly CTL019) has produced remission in up to 90% of pediatric patients with r/r ALL in a single-center trial^25^ and in 81% in a global, multicenter, phase 2 trial^26^. Additional CAR T cell therapies being evaluated in the pediatric r/r ALL setting have shown similar response rates of 70–93% in phase 1 trials, but the durations of response and recommendations for SCT after CAR therapy varied for each treatment^27,28^. Based on the results from the phase 2 trial, tisagenlecleucel was approved by the US Food and Drug Administration in 2017 for the treatment of pediatric or young adult patients with B cell ALL (B-ALL) that is refractory to treatment or that is in second or greater relapse.

In this article, we summarize four cases of pediatric and adolescent/young adult patients with r/r ALL who benefited from CAR T cell therapy, with a focus on the patient experiences and treatment regimens leading up to CAR T cell therapy and the management of toxicity postinfusion^29,30^.

### Patient cases

#### Patient 1. CAR T cell therapy for relapse after prior SCT

Currently, allogeneic SCT is the preferred treatment option for eligible patients who have a high risk of relapse following initial treatment with chemotherapy^10^; however, approximately one third of patients undergoing SCT will relapse^31^. Once salvage chemotherapy and SCT have failed, there are few curative treatment options for these patients. Case 1 is an example of CAR T cell therapy following post-SCT relapse.

Patient 1 was aged 4 years at the time of initial diagnosis of National Cancer Institute standard-risk B-ALL in July 2002 who presented with a WBC count of 4900/µL and non-informative cytogenetics. There was no evidence of CNS or testicular disease. The patient was treated with Pediatric Oncology Group intermediate-risk therapy 9905 regimen C^32^ and completed treatment in January 2005. Four years later, at 11 years of age, the patient experienced a late, isolated BM relapse and was treated according to Children’s Oncology Group (COG) protocol AALL0433^1^. Following conditioning with cyclophosphamide and total body irradiation, the patient received matched-sibling SCT in January 2010. The patient experienced no graft-vs-host disease and was able to discontinue immunosuppression within 60 days.

Unfortunately, the patient experienced a second relapse 3 years following SCT in 2013, again isolated to the BM. The patient underwent a 4-drug reinduction treatment regimen but had highly refractory disease with persistence of 70% peripheral blasts. As a result, the patient was referred for CAR T cell therapy.

The patient underwent successful leukapheresis and, during the CAR T cell manufacturing process, received bridging chemotherapy. Owing to the patient’s highly chemoresistant disease, intensive salvage chemotherapy was required for disease control with 5-day pulses of etoposide and cyclophosphamide plus prophylactic intrathecal therapy to prevent concomitant CNS recurrence. The patient was reinfused with anti-CD19 CAR T cells in May 2013 at 15 years of age. Five days after infusion, the patient developed fever and myalgias, which progressed quickly to grade 3 cytokine release syndrome (CRS)^29^ based on hypotension requiring high-dose infusion of norepinephrine^30^ and hypoxia requiring bilevel positive airway pressure. Upon transfer to the pediatric intensive care unit (PICU), the patient received CRS rescue therapy with tocilizumab 8 mg/kg and intravenous methylprednisolone 1 mg/kg twice daily for 2 days^29^. The patient improved over the next 5 days and was discharged from the hospital at day 18. The patient remains in remission 5 years after infusion.

#### Patient 2. CAR T cell therapy following BM and CNS relapse post-SCT and donor lymphocyte infusion

Intrathecal chemotherapy is routinely given as part of treatment for ALL to treat and/or prevent seeding of leukemic blasts in the CNS. Nonetheless, CNS relapse affects 20–25% of patients^9,13,14^. In addition to systemic chemotherapy that penetrates the CNS, intrathecal therapy and cranial irradiation increase the rates of CR in patients with CNS disease at relapse. However, the toxicity associated with use of cranial radiation in children aged <5 years with CNS relapse is significant^33^. Despite multimodal therapy, long-term outcomes of patients with CNS relapse, particularly in close proximity to treatment, remain poor^9^.

Patient 2 was originally diagnosed with Ph+ B-ALL in May 2005 at 5 years of age. At the time of diagnosis, his WBC count was 75,000/µL and no extramedullary disease was evident. The patient was initially treated per COG protocol AALL0232^34^. In October 2009, the patient experienced an isolated BM relapse and was treated according to COG first-relapse trial protocol AALL01P2^1,35^ with 3 months of intensive chemotherapy including imatinib, followed by a 10/10-matched, unrelated-donor SCT.

Unfortunately, the patient experienced a combined BM and CNS relapse <1 year after SCT. The patient was treated with multiagent chemotherapy (vinorelbine, topotecan, thiotepa, clofarabine, dexamethasone, and intrathecal cytarabine) followed by donor lymphocyte infusion and achieved another short-lived remission. Within weeks of the donor lymphocyte infusion, disease recurred in the BM and CNS; thus, treatment continued with cyclophosphamide/etoposide and dasatinib, and the patient achieved a fourth remission. The patient continued to be treated on and off for multiple BM, CNS, and combined relapses. Treatment during this time also included nilotinib. Owing to the persistent recurrence of CNS disease, the patient was referred for anti-CD19 CAR T cell therapy in 2012.

Between T cell collection and CAR T cell infusion, the patient’s CNS disease was treated with twice weekly intrathecal chemotherapy until the patient’s cerebrospinal fluid showed clearing of lymphoblasts. Dasatinib was continued for systemic control. In 2013, the patient underwent CAR T cell infusion and had very mild CRS that did not require intervention or transfer to the PICU. The patient did develop grade 2 encephalopathy due to some word finding issues and confusion for which he received supportive care only. The confusion resolved within 48 hours and he was discharged soon after. He remains in remission 4 years after treatment.

#### Patient 3. CAR T cell therapy for primary refractory Ph-like ALL

Although the risk factors for primary induction failure are poorly understood, children and young adults with Ph-like ALL are at higher risk for induction failure and persistent minimal residual disease (MRD)^13,36,37^. Ph-like ALL is uncommon in young children but increases in prevalence with age and is found in approximately 25% of adolescents and young adults^37,38^. Owing to poor response to initial therapy, many patients with Ph-like ALL are referred for SCT. However, current standards for pediatric and adolescent patients are to undertake SCT only if MRD is <0.1%, which can be difficult to obtain in this patient population^39–41^.

Patient 3 was initially diagnosed in 2014 at 16 years of age. At diagnosis, the patient’s WBC count was >200,000/µL, supporting a diagnosis of very-high-risk pre-B-ALL. The patient was treated according to COG high-risk ALL protocol AALL1131^1^. Molecular testing further characterized the patient’s disease as *EBF1-PDGFRB1*+ (Ph-like ALL); as a result, dasatinib was added to induction chemotherapy on day 15. At the end of induction, the patient’s BM demonstrated 67% blasts, meeting the criteria for primary induction failure (>25% leukemic blasts). At day 29 of consolidation, blasts remained at 65%, and at the end of consolidation, MRD was still 0.13%, making the patient ineligible for SCT.

At the time of diagnosis of high-risk ALL, anticipating highly chemotherapy-resistant leukemia and disease persistence, the patient’s team arranged for the patient to undergo leukapheresis to collect T cells after induction chemotherapy when the BM blast count was 67%. The decision to proceed to leukapheresis early in the course of therapy was made to maximize the collection of sufficient T cells for CAR T cell manufacture. Treatment continued with COG AALL1131-like therapy with cycles of high-dose methotrexate during CAR T cell manufacturing, as the patient continued with only MRD-level disease. More intensive salvage therapy would have been considered if the patient had increasing peripheral blasts requiring improved disease control. Anti-CD19 CAR T cells were reinfused, and the patient achieved an MRD-negative CR. The patient had grade 1 CRS with fever, fatigue, and myalgias, requiring a 1-week hospitalization.

Nine months postinfusion, a routine surveillance BM was morphologically negative but was MRD positive at a level of 0.8% with CD19-negative blasts. CD19-negative recurrence is a known mechanism of CART-19 escape and accounts for about 23–28% of events^26,42^. MRD surveillance post-CAR T should only be done at a center that can assess for CD19-negative blasts. The patient received a cycle of CD22 targeted therapy with inotuzumab^43^, managed to obtain a second remission, and proceeded to a matched, unrelated-donor SCT while still in CR. The patient remains in remission almost 2 years later.

#### Patient 4. CAR T cell therapy in a patient with Down syndrome and relapsed ALL

Children and adolescents with Down syndrome are at increased risk for developing ALL^44^ and are more likely to experience poor outcomes following relapse^45^. Studies have shown poor outcomes related to leukemia relapse as well as toxicity from reinduction therapy and/or SCT conditioning regimens^46^. This is a particularly vulnerable group in need of newer targeted therapies to reduce relapse and/or treatment-related toxicities.

Patient 4 had trisomy 21 and was diagnosed at 8 years of age with National Cancer Institute standard-risk ALL. As an infant, the patient had a history of ventricular and atrial septal defects, which were successfully surgically repaired. Initial treatment was with COG standard-risk protocol AALL0932 for patients with Down syndrome, and the day 29 BM was MRD <0.01%^1^. The patient had minimal toxicity from therapy but, 4 years after completing treatment, relapsed at 14 years of age. Despite responding well to standard first-line relapse therapy, the patient experienced a second relapse. The health-care team determined that the toxicity risk for SCT or ongoing cytotoxic chemotherapy was unacceptably high. The patient was instead referred for CAR T cell therapy.

The patient successfully underwent leukapheresis and, while the CAR T cells were manufactured, received bridging chemotherapy with low-dose maintenance-like ALL therapy^34^ to try to avoid toxicity. CAR T cell infusion occurred in May 2016. On day 5 postinfusion, the patient was admitted with mild CRS; however, on day 8, the patient had a seizure requiring initiation of levetiracetam, which is our institutional norm for CAR T cell patients. On day 28, the patient was found to be in an MRD-negative remission and the levetiracetam was discontinued without further event. The patient remains in remission 29 months later.

## Discussion

**Fig. 2:**
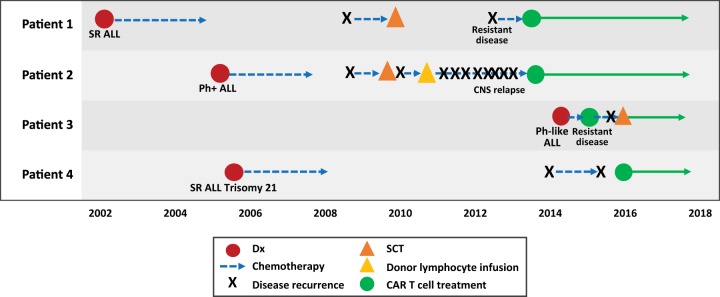
Timeline of patient journey to CAR T cell therapy. The journey of CAR T cell therapy for each individual patient. All patients proceeded to CAR T cell therapy after failing conventional salvage treatments or presenting with refractory disease. B-ALL B cell acute lymphoblastic leukemia, CAR chimeric antigen receptor, CNS central nervous system, Dx diagnosis, Ph Philadelphia chromosome, SCT stem cell transplant, SR standard risk

The four cases presented here represent different aspects of r/r ALL that are difficult to treat and are associated with poor outcomes: relapse after SCT, multiple CNS relapses, primary refractory ALL, and relapsed ALL in a pediatric patient with Down syndrome (Fig. 2). In all four cases, CAR T cell therapy was a new opportunity for patients whose primary alternative was palliative care. The patients remain in remission ranging from 2 to 5 years.

Despite significant progress in clinical outcomes of ALL, children and adolescents with r/r ALL represent an unmet need. Patients with ALL that is refractory to standard chemotherapy regimens, who have relapsed disease, and who are ineligible for SCT or experience relapse after SCT have limited treatment options. Further confounding some patients’ prognoses can be other factors, including disease cytogenetics and comorbidities, such as Down syndrome, or prior toxicities from therapy. Immunotherapies are a promising new direction, in part, because directly targeting the leukemic cell through an immune mechanism bypasses chemoresistance, cytogenetics, and other patient factors. Tisagenlecleucel is a CAR T cell therapy that reprograms patients’ T cells to identify and destroy CD19-expressing B cells. The success of this therapy has been reported in a single-center trial^42^ and, more recently, in a global, multicenter trial^26^. The integration of CAR T cell therapy into existing treatment paradigms remains to be fully established.

It is likely that CAR T cell therapy will be adopted in place of repeated SCTs for patients who have relapsed following SCT, as outcomes following second (or greater) SCT are very poor^47,48^. A retrospective study of the Center for International Blood and Marrow Transplant Research registry found that 3-year survival in patients after first SCT was 63%, but 29% relapsed^31^. Outcomes of patients who relapse following SCT are particularly poor; 5-year overall survival in patients who receive SCT in second remission is 40%, while 5-year overall survival in patients in third or greater remission is only 33%^49^. In a recently published phase 2, global study of CAR T cell therapy, overall response rates were comparable between patients who had previously undergone SCT and those who had not^26^. Although long-term data are not yet available, 1-year overall survival in this trial was 76%, suggesting that CAR T cell therapy may be an effective treatment for patients who relapse following SCT. Patient 1 is an example of the success of CAR T cell therapy following post-SCT relapse. In the future, CAR T cell therapy may also be a consideration in place of SCT for young pediatric patients who may have significant long-term toxicities from total body irradiation, cranial radiation, intensive chemotherapy, or genetic predisposition syndromes that make them more susceptible to severe toxicities from cytotoxic therapy.

Given advances in the detection of submicroscopic disease, a marker for elevated risk of relapse, and persistent disease with MRD, CAR T cell therapy may be an option earlier in treatment for patients with resistant disease or those with the highest risk of recurrence. Patient 3 is an example of MRD detection of chemoresistant disease and early transition to CAR T cell therapy. MRD positivity in refractory patients as well as those with recurrent disease may be an important method to identify those who will benefit from CAR T cell therapy. In appropriate patients, CAR T cell therapy may provide a bridge to SCT, potentially avoiding complications associated with cytotoxic chemotherapy, such as life-threatening infection. Thus far, many of the patients at our institution who have undergone CAR T cell therapy without prior SCT have chosen to not undergo consolidative SCT after CAR T cell therapy due to the very good outcomes with CAR T cell therapy alone. However, no randomized trials exist comparing CAR T cell therapy and SCT.

Much like in the settings of post-SCT recurrence and primary chemoresistance, CAR T cell therapy may be a much-needed alternative treatment for patients with recurrent extramedullary leukemia, including relapsed CNS disease, as discussed in the case of Patient 2. Although isolated extramedullary relapse has a better prognosis than BM recurrence, CNS leukemia at diagnosis is an independent risk factor for relapse and inferior outcome^50^; SCT is also less effective in patients with CNS involvement at relapse. Thus CAR T cell therapy may be a superior option for patients with CNS relapse, including those with low-level disease and cranial nerve involvement. Importantly, CAR T cell therapy does not require further radiation therapy in patients who may be nearing the maximum tolerated dose of cranial radiation or in whom it would be neurotoxic. Data thus far in patients with CNS relapse treated with CAR T cell therapy indicate positive outcomes. In an analysis of 53 patients with r/r ALL who received tisagenlecleucel, 12 had previous CNS relapse. Of those patients, 8 (67%) experienced a CR for a median of 8 months (range, 3–22 months) with no CNS relapse. Importantly, 98% of infused patients evaluated had detectable tisagenlecleucel in their cerebral spinal fluid^51^.

Finally, CAR T cell therapy may be particularly valuable for vulnerable patient populations, including children and adolescents with Down syndrome, chromosomal breakage syndromes, Li Fraumeni syndrome, or toxicities from prior therapy that excluded SCT. This is an important group for getting back into remission after relapse given the toxicities associated with salvage chemotherapy and SCT preparative regimens. In particular, patients with Down syndrome have increased relapse rates and fare particularly poorly following relapse, with 3-year survival of 10–25%^52^. In a phase 2, global trial of CAR T cell therapy, 6 patients with Down syndrome were infused with tisagenlecleucel with no other treatment modifications. Overall response rates (including CR and CR with incomplete hematologic recovery) in this patient subset were comparable to those of patients who did not have Down syndrome^26^. Rates of adverse events were also similar^53^.

It is important to note that, although each of the patients discussed here benefited from CAR T cell therapy, there are some significant limitations to the procedure. In a small number of patients, adequate numbers of T cells cannot be collected, often due to the intensive lymphocytotoxic chemotherapy they have received or if they are within months of receiving SCT. One strategy is to collect T cells early in treatment if there are indications that a patient may be at high risk for chemoresistant disease, as in the case of Patient 3 with Ph-like disease and persistent blasts through induction. In addition to inadequate T cell collection, the manufacturing process can fail. In a recently published phase 2, global study, product-related issues occurred in 7 of the 92 enrolled patients^26^.

In the days and weeks immediately after CAR T cell infusion, CRS and neurological toxicities were common; most patients treated with CAR T cells were hospitalized, and some required intensive care unit–level care, including intubation and vasopressors. The cases reviewed here had expected toxicities within the typical side effect profile of tisagenlecleucel, and all were manageable. Very clear guidelines now exist for the management of CRS^29^. Administration of the interleukin-6 pathway inhibitor tocilizumab shortened the duration of CRS and has been given to patients with severe symptoms^42^. Corticosteroids have also been administered, but the anti-inflammatory impact on CAR T cell efficacy has not been fully evaluated.

Neurological events were also common following CAR T cell infusion, most frequently in conjunction with severe CRS or shortly after its resolution. The most common events reported in the global trial were encephalopathy, confusion, delirium, tremor, agitation, and somnolence and were managed with supportive care only. In most cases, neurological symptoms were transient and resolved within days to weeks of infusion^26^. It is reasonable to consider seizure prophylaxis for patients with a history of seizure disorder, prior traumatic CNS event such as stroke or hemorrhage, or CNS disease^54,55^. Most experts recommend levetiracetam for 30–60 days following tisagenlecleucel administration.

A final consideration following CAR T cell therapy is the long-term consequences of harboring virally modified T cells in a patient’s body. CAR T cells have been reported to be detectable in the peripheral blood of treated patients for up to 2 years, and this timeframe will likely extend as longer follow-up of infused patients is completed^42^. Because destruction of B cells is an on-target effect of CAR T cells, patients who are successfully treated can have B cell aplasia for years, perhaps indefinitely, putting them at increased risk for infection and potentially other yet unknown adverse effects. Immunoglobulin replacement is one strategy to prevent infection, but there are no long-term studies on the impact of persistent B cell aplasia. In addition, the long-term effects of genetically modified T cells and the possibility of integration-mediated oncogenesis remain to be determined.

Despite revolutionary responses in high-risk patients, relapse does occur post-CAR T cell therapy. There are several known mechanisms by which this may occur. First, loss of engineered T cells: it is not known the exact length of time necessary for a durable response/cure; however, it is clear that shorter persistence is associated with a higher risk of relapse^56,57^. Accordingly, patients should be monitored for loss of engineered T cells, which in the case of CD19-directed therapy can be accomplished by monitoring for recovery of normal CD19+ B cells and/or bone marrow hematogones. A potential solution to this problem is re-infusion of additional doses of tisagenlecleucel. Early B cell recovery postinfusion (1–3 months) may be a sign of immune-medicated rejection of tisagenlecleucel, and a second-generation fully humanized anti-CD19 CAR T cell has shown promise in both the CD19+ relapse post CAR T cells and the CD19-naive patient populations^58^. A second mechanism of relapse is CD19-negative relapse. In this case, the leukemic blasts no longer express the necessary antigen, and no amount of persistent CAR19 T cells will be effective. Alternative therapies are required in this case, whether other antigen-directed approaches (such as inotuzumab ozogamicin^43^ directed against CD22) or more broadly cytotoxic chemotherapy. Additionally, phase 1/2 trials of CAR T cells targeted against CD22 are underway, as well as preclinical work to identify other potential leukemia protein targets such as CRLF2, mixed lineage leukemia (MLL), and CD123. Furthermore, bivalent CARs targeting CD22 and CD19 are currently in clinical development^59^.

**Fig. 3:**
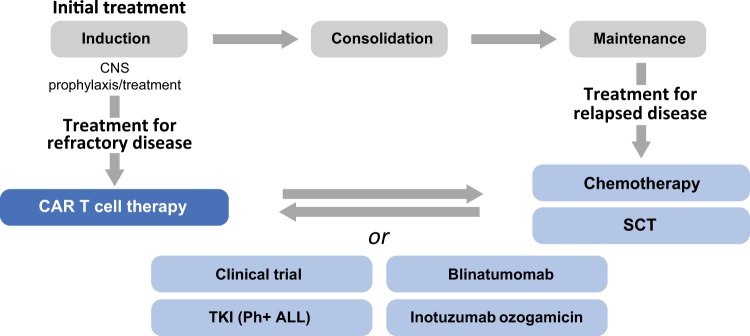
Diagram of treatment paradigms that include CAR T cell therapy. Integration of CAR T cell therapy into the treatment algorithm for patients with B-ALL. B-ALL B cell acute lymphoblastic leukemia, CAR chimeric antigen receptor, CNS central nervous system, Ph Philadelphia chromosome, SCT stem cell transplant, TKI tyrosine kinase inhibitor

In conclusion, CAR T cell therapy is an important new option for pediatric and young adult patients. CAR T cell therapy may be integrated into the treatment paradigm at multiple points. For patients with primary refractory disease, CAR T cell therapy may be a first-line salvage option. In other cases, CAR T cell therapy may be most appropriate after a patient experiences disease recurrence following salvage chemotherapy and/or SCT. Finally, CAR T cell therapy may be an important option to prepare patients for successful SCT (Fig. 3). The efficacy of CAR T cell therapy in diverse patients is an important advance in the treatment of pediatric and adolescent patients with r/r ALL.
